# Psychological Impact and Quality of Life in Chronic Psoriasis Patients: A Mixed-Methods Study Combining a Validated Researcher-Developed Questionnaire With In-Depth Patient Narratives

**DOI:** 10.7759/cureus.113137

**Published:** 2026-07-22

**Authors:** Sudeep Jayaram, Neethu S, Romesh Bagde

**Affiliations:** 1 Geriatrics, Aneurin Bevan University Health Board, Cardiff, GBR; 2 Physician, Valluvanad Hospital, Ottapalam, IND; 3 Psychiatry and Behavioral Sciences, N.K.P. Salve Institute of Medical Sciences and Research Centre and Lata Mangeshkar Hospital, Nagpur, IND

**Keywords:** chronic psoriasis, dermatology, mixed-methods study, psychological impact, psychosocial burden, quality of life

## Abstract

Background: Psoriasis is a chronic inflammatory skin disease associated with a significant physical and psychosocial burden. It adversely affects not only the skin but also psychological well-being, social functioning, and overall quality of life. Although quantitative assessment tools provide valuable estimates of disease burden, they do not fully capture patients' lived experiences. This concurrent mixed-methods study aimed to explore the impact of chronic psoriasis on psychological well-being, quality of life, and coping strategies among affected individuals.

Methods: A concurrent cross-sectional mixed-methods study was conducted among 60 adults with chronic psoriasis attending a tertiary care dermatology clinic. Disease severity was assessed using the Psoriasis Area and Severity Index (PASI) and categorized as mild, moderate, or severe. Quantitative data were collected using a researcher-developed questionnaire assessing quality of life, psychological impact, social functioning, coping strategies, and patient-reported support needs. The questionnaire was developed following a review of the published literature, underwent expert content validation by a three-member multidisciplinary panel (Content Validity Index = 0.90), and demonstrated good internal consistency (Cronbach's α = 0.80). A purposive subsample of 20 participants subsequently underwent in-depth semistructured interviews, and qualitative data were analyzed using inductive thematic analysis.

Results: Among the 60 participants, PASI-based disease severity assessment classified 28 (46.67%) participants as having moderate psoriasis and 18 (30.0%) as having severe psoriasis. Moderate-to-severe impairment in quality of life was observed in 48 (80.0%) participants, while psychological distress was present in 44 (73.3%), including moderate distress in 17 (28.3%) and severe distress in nine (15.0%) participants. Social interaction was adversely affected in 39 (65.0%) participants, and work performance was impaired in 34 (56.7%). Feelings of embarrassment were reported by 41 (68.3%) participants, anxiety by 36 (60.0%), and low self-esteem by 33 (55.0%). Qualitative analysis identified four major themes: perceived stigma, emotional burden, social withdrawal, and coping strategies.

Conclusion: Chronic psoriasis was associated with substantial impairment in quality of life and significant psychological distress. Integrating quantitative findings with qualitative patient narratives provided a more comprehensive understanding of the psychosocial burden experienced by affected individuals. The findings highlight the importance of incorporating psychosocial assessment, patient education, and appropriate psychological support into comprehensive dermatological care.

## Introduction

Psoriasis is a chronic inflammatory skin disorder caused by an immune dysregulation resulting in erythematous scaly plaques with a relapsing-remitting course, affecting all age groups. It is increasingly considered a systemic disease affecting multiple organ systems beyond the skin [1,2]. Due to its chronicity, visibility, and unpredictable nature of the disease course, psoriasis poses a significant psychological burden on patients’ overall health and well-being [3]. The psychosocial impact of psoriasis in terms of embarrassment, stigmatization, and impaired self-esteem is commonly seen, especially among patients with lesions affecting visible parts of their bodies, such as the face, scalp, and hands [4,5].

Thus, it effectively causes psychological comorbidities in the form of anxiety, depression, and social isolation, as well as impaired quality of life (QoL) [6,7]. Hence, QoL assessment has become an integral part of comprehensive psoriasis management and treatment [8]. Patient-reported outcome measures of assessing the impact of dermatological diseases on daily psychological well-being are widely used. However, they may not do justice to the subtlety of patients’ lived experience expressing adaptation to emotional distress, coping strategies used, and tacit social stigmatization of nonintentional chronic skin disease [9]. Qualitative research methods, especially in-depth interviews, help provide context to quantitative assessments [10].

There is growing recognition that integrating quantitative and qualitative research methods provides a more comprehensive understanding of the psychosocial burden associated with chronic dermatological diseases by combining measurable outcomes with patients' lived experiences [11,12]. Although several studies have examined the psychological and quality-of-life impact of psoriasis using quantitative or qualitative approaches, comparatively few have integrated both methodologies within a single study to provide complementary insights into patients' experiences. Therefore, the present study employed a concurrent mixed-methods design to evaluate the psychological well-being and quality of life of patients with chronic psoriasis by combining a researcher-developed quantitative questionnaire with in-depth qualitative patient narratives.

## Materials and methods

Study setting and design

This cross-sectional concurrent mixed-methods study was conducted in the Department of Dermatology of a tertiary care teaching hospital over a period of four months. Quantitative and qualitative data were collected concurrently from the same study population and integrated during the interpretation stage to obtain a comprehensive understanding of the psychological impact and quality of life among patients with chronic psoriasis. The quantitative component assessed patient-reported psychosocial outcomes, whereas the qualitative component explored participants' lived experiences, emotional burden, perceived stigma, coping strategies, and support needs through in-depth semistructured interviews.

A researcher-developed questionnaire was used because the objectives of the present study extended beyond the assessment of quality of life alone and included multiple context-specific domains such as psychological impact, social functioning, coping strategies, and patient-reported support needs, which could not be comprehensively captured using a single standardized instrument. The questionnaire was specifically developed to address the objectives of the present mixed-methods study (see the Appendix).

Study approval was obtained from the Institutional Ethics Committee (IEC) of N.K.P. Salve Institute of Medical Sciences and Research Centre and Lata Mangeshkar Hospital, Nagpur, Maharashtra, India (approval no. NKPSIMS & RC and LMH/IEC/2/2026; approved on January 29, 2026). Written informed consent was obtained from all participants prior to enrolment.

Selection Criteria

Adult patients aged 18 years or older with clinically diagnosed chronic psoriasis (disease duration greater than or equal to six months) who attended the dermatology outpatient department during the study period were eligible for inclusion. Participants were required to understand the study procedures and provide informed consent.

Patients with a documented psychiatric illness preceding the diagnosis of psoriasis, those receiving treatment for a major psychiatric disorder at the time of recruitment, patients presenting with acute psoriasis requiring urgent medical management, individuals with other chronic dermatological disorders likely to independently influence quality of life, and those unwilling or unable to participate in the study were excluded.

Sample Size

For the quantitative component, the sample size was determined with reference to previously published hospital-based studies evaluating the psychological impact and quality of life among patients with chronic psoriasis using questionnaire-based assessment methods [1]. Considering the exploratory nature of the study, the feasibility of participant recruitment during the study period, and the objectives of the study, a total of 60 participants were included.

For the qualitative component, a purposive subsample representing diverse demographic and clinical characteristics was selected for in-depth interviews. The qualitative sample size was guided by the principle of thematic saturation rather than statistical power. Based on previous qualitative dermatology research, thematic saturation was anticipated within approximately 15-20 interviews and was achieved after interviewing 20 participants, at which point no substantially new themes emerged.

Data collection

Participants were recruited consecutively from the dermatology outpatient clinic. Demographic and clinical information was collected using a structured case record form, including age, sex, marital status, educational status, occupation, disease duration, clinical type of psoriasis, anatomical sites involved, previous treatment history, visible skin involvement, and family history of psoriasis. Disease severity was assessed by the treating dermatologist using the Psoriasis Area and Severity Index (PASI), which evaluates lesion erythema, induration, scaling, and body surface area involvement. Patients were categorized as having mild (PASI <7), moderate (PASI 7-12), or severe psoriasis (PASI >12) [13]. Quantitative data were collected using a structured researcher-developed questionnaire designed to assess quality of life, psychological impact, social functioning, coping strategies, and patient-reported support needs among patients with chronic psoriasis.

The questionnaire was developed following an extensive review of published literature addressing the psychosocial burden and quality-of-life impact of psoriasis and was based on domains commonly explored in previous studies [2,9]. Content validity was established through evaluation by a three-member multidisciplinary expert panel comprising specialists from the Departments of Dermatology, Psychiatry, and Community Medicine. The questionnaire demonstrated excellent content validity with a Content Validity Index of 0.90 [14]. Internal consistency reliability was assessed using Cronbach's alpha, which demonstrated good reliability (Cronbach's α = 0.80). The finalized questionnaire was approved by the IEC before being used for data collection.

For the qualitative component, purposively selected participants underwent face-to-face semistructured interviews using a predeveloped interview guide. Interviews were conducted in a private setting by the principal investigator, lasted approximately 30-45 minutes, and were audio-recorded with participants' written consent before being transcribed verbatim. Field notes were maintained throughout the interviews to document contextual observations and nonverbal cues that aided interpretation.

Qualitative data analysis

Qualitative data were analyzed using inductive thematic analysis following the six-phase framework described by Braun and Clarke [15]. Two investigators independently reviewed the interview transcripts to familiarize themselves with the data and generated initial codes. Coding discrepancies were resolved through discussion until consensus was achieved. Related codes were subsequently grouped into broader categories and overarching themes that reflected participants' psychosocial experiences.

An audit trail documenting coding decisions and theme development was maintained throughout the analytical process to enhance methodological transparency. To improve the credibility of the findings, member checking was performed with a subset of participants to confirm that the emerging themes accurately reflected their lived experiences. Investigator reflexivity was maintained throughout data analysis by acknowledging potential researcher assumptions during coding and interpretation.

Statistical analysis

Quantitative data were entered into Microsoft Excel (Microsoft Corporation, Redmond, WA) and analyzed using IBM Statistical Package for the Social Sciences, version 23 (IBM Corp., Armonk, NY). Continuous variables were summarized as mean ± standard deviation, whereas categorical variables were presented as frequencies and percentages. Qualitative interview transcripts were analyzed using inductive thematic analysis as described above. Finally, quantitative and qualitative findings were integrated during interpretation through methodological triangulation to provide a comprehensive understanding of the psychological burden, quality of life, coping strategies, and lived experiences of patients with chronic psoriasis.

## Results

In total, there were 60 individuals diagnosed with chronic psoriasis who were involved in this study (i.e., overall study sample). All participants were evaluated quantitatively; however, 20 of those individuals were selected (purposely) to be interviewed in-depth qualitatively (from the overall sample). The demographic characteristics of the study participants are summarized in Table 1. The mean age of participants was 41.6 ± 10.2 years. Most participants were men, married, and employed, and had completed at least secondary education.

**Table 1:** Demographic characteristics of study participants Values are presented as mean ± standard deviation or n (%)

Parameter	Value (n = 60)
Mean age (years)	41.6 ± 10.2
Gender (male/female)	34/26
Married	42 (70%)
Employed	38 (63.3%)
Education ≥ secondary	45 (75%)

Table 2 presents the clinical characteristics of psoriasis for the individuals who participated in the study. The average disease duration was 6.8 ± 4.1 years, and the most common clinical type was chronic plaque psoriasis. A large number of patients in this study had visible affected areas, such as their scalp or nails.

**Table 2:** Clinical characteristics of psoriasis Values are presented as mean ± standard deviation or n (%)

Parameter	Value
Mean disease duration (years)	6.8 ± 4.1
Chronic plaque psoriasis	46 (76.6%)
Scalp involvement	32 (53.3%)
Nail involvement	18 (30.0%)
Visible area involvement	40 (66.7%)

The distribution of disease severity, as classified by the PASI, along with the extent of quality-of-life impairment, is presented in Table 3. Moderate psoriasis was observed in 28 participants (46.67%), while severe psoriasis was observed in 18 participants (30.0%). With respect to quality of life, a similar pattern emerged: a large proportion of subjects reported a moderate-to-severe impairment.

**Table 3:** Distribution of psoriasis severity and quality-of-life impairment among study participants (n = 60) Values are presented as number (%) QoL: quality of life

Category	Value
Mild psoriasis	14 (23.3%)
Moderate psoriasis	28 (46.67%)
Severe psoriasis	18 (30.0%)
Mild QoL impairment	12 (20.0%)
Moderate QoL impairment	26 (43.3%)
Severe QoL impairment	22 (36.6%)

Table 4 presents the distribution of psychological distress according to disease duration. The proportion of participants with moderate-to-severe psychological distress increased with longer disease duration, rising from 19.0% among patients with disease duration of less than five years to 50.0% among those with disease duration of 5-10 years and 64.7% among patients with disease duration exceeding 10 years.

**Table 4:** Psychological distress distribution by disease duration Values are presented as numbers (row percentage), except for the total row, where percentages represent the overall study population

Disease duration	Normal, n (%)	Mild, n (%)	Moderate, n (%)	Severe, n (%)	Total
<5 years (n = 21)	10 (47.6)	7 (33.3)	3 (14.3)	1 (4.8)	21
5-10 years (n = 22)	5 (22.7)	6 (27.3)	7 (31.8)	4 (18.2)	22
>10 years (n = 17)	1 (5.9)	5 (29.4)	7 (41.2)	4 (23.5)	17
Total (n = 60)	16 (26.7)	18 (30.0)	17 (28.3)	9 (15.0)	60

The effect of psoriasis on emotional and daily functioning is illustrated in Table 5. Social interaction and work performance were among the most frequently affected domains. In terms of emotional response, subjects frequently reported feelings of embarrassment, anxiety, and low self-esteem.

**Table 5:** Impact on daily activities and emotional responses Values are presented as numbers and frequencies

Parameter	Value, n (%)
Social interaction affected	39 (65%)
Work performance affected	34 (56.6%)
Sleep quality affected	28 (46.6%)
Physical activity affected	26 (43.3%)
Embarrassment	41 (68.3%)
Anxiety	36 (60%)
Low self-esteem	33 (55%)
Depressive feelings	27 (45%)

According to Table 6, the most frequently reported coping strategy was social avoidance (29, 48.3%), followed by acceptance (25, 41.6%), positive reframing (22, 36.6%), and seeking social support (18, 30.0%). These findings suggest that patients commonly relied on both avoidance-based coping (social withdrawal) and adaptive coping strategies, including acceptance, positive cognitive reframing, and support-seeking, to manage the psychosocial burden of chronic psoriasis. Regarding patient-reported needs, a holistic care approach (47, 78.3%), better disease education (44, 73.3%), and psychological counseling (38, 63.3%) were the most frequently identified requirements.

**Table 6:** Coping strategies and patient-reported needs Values are presented as n (%)

Parameter	Value
Social avoidance	29 (48.3%)
Positive reframing	22 (36.6%)
Seeking social support	18 (30%)
Acceptance	25 (41.6%)
Psychological counseling	38 (63.3%)
Better disease education	44 (73.3%)
Support groups	31 (51.6%)
Holistic care approach	47 (78.3%)

Qualitative analysis identified four major themes: perceived stigma, emotional burden, social withdrawal, and coping strategies. Interview transcripts were independently coded by two investigators using an inductive thematic analysis approach, and discrepancies were resolved through discussion until consensus was achieved. A summary of the identified themes and representative participant quotations is presented in Table 7. Overall, the qualitative findings complemented the quantitative results by providing deeper insight into the psychosocial challenges experienced by patients with chronic psoriasis and the coping mechanisms adopted in daily life.

**Table 7:** Summary of qualitative themes identified from the thematic analysis

Theme	Description	Representative quotation
Perceived stigma	Participants frequently described feeling stigmatized because of the visible nature of their skin lesions, reporting embarrassment, fear of negative social judgment, and perceived social discrimination	"People often stared at my skin and avoided touching me. It made me feel embarrassed to go out in public"
Emotional burden	Participants expressed persistent anxiety, frustration, reduced self-confidence, and emotional distress related to the chronic and unpredictable course of psoriasis	"Living with psoriasis has affected my confidence. I constantly worry about what others think about my appearance"
Social withdrawal	Several participants reported avoiding social gatherings, limiting interpersonal interactions, and reducing participation in family and community activities because of fear of stigma and unwanted attention	"I gradually stopped attending family functions because I felt uncomfortable answering questions about my skin"
Coping strategies	Participants adopted various coping strategies, including acceptance, positive reframing, seeking family support, adherence to treatment, and, in some cases, social avoidance	"Over time, I accepted my condition and learned to focus on my treatment rather than worrying about other people's opinions"

## Discussion

The present concurrent mixed-methods study evaluated the psychological impact and quality of life among patients with chronic psoriasis by integrating quantitative assessment with qualitative patient narratives. The findings demonstrated that chronic psoriasis is associated with substantial impairment in quality of life, psychological distress, and social functioning, reinforcing the concept that psoriasis is a multidimensional disease extending well beyond its cutaneous manifestations. In the present study, 80.0% of participants experienced moderate-to-severe quality-of-life impairment, while 73.3% reported psychological distress, indicating a considerable psychosocial burden. These observations are consistent with previous studies by Bulat et al. [1], Bhosle et al. [2], and Agarwal et al. [5], all of whom reported that psoriasis significantly compromises emotional well-being, interpersonal relationships, occupational functioning, and overall quality of life. Our findings further support the growing recognition that routine assessment of psychosocial outcomes should complement clinical evaluation in patients with chronic psoriasis.

Psychological distress increased with longer disease duration in the present study, with moderate-to-severe distress being more frequently observed among patients who had lived with psoriasis for more than 10 years. Similar associations between prolonged disease duration and increased anxiety, depression, and emotional distress have been reported by Sarkar et al. [16], Frede et al. [17], and Jankowiak et al. [18]. However, unlike studies that have primarily focused on standardized patient-reported outcome measures, the present study additionally explored patients' lived experiences through qualitative interviews, providing a contextual understanding of how persistent disease influences social participation, interpersonal relationships, and emotional adaptation over time.

Social functioning emerged as one of the most affected domains, with reduced social interaction, impaired work performance, embarrassment, anxiety, and low self-esteem commonly reported by participants. These findings are in agreement with previous literature demonstrating that visible skin lesions frequently lead to stigmatization, altered body image, social discrimination, and reduced self-confidence among individuals with psoriasis [18,19]. While these psychosocial consequences have been consistently documented, the qualitative narratives in the present study further illustrated how patients gradually modify their daily behaviors, avoid social situations, and develop individualized coping mechanisms in response to perceived stigma, thereby adding greater depth to the quantitative observations.

The qualitative analysis identified four overarching themes: perceived stigma, emotional burden, social withdrawal, and coping strategies. These themes closely resemble those reported by Meneguin et al. [9] and Zhong et al. [19], who described stigma, emotional suffering, and social isolation as central experiences among patients with psoriasis. However, the present study additionally highlighted the coexistence of both adaptive coping strategies, such as acceptance and positive reframing, and maladaptive coping strategies, particularly social avoidance. This finding suggests that patients employ diverse psychological responses while adjusting to the chronic nature of the disease and underscores the importance of individualized psychosocial support alongside dermatological management [20].

Participants also identified psychological counseling, improved disease education, and a holistic care approach as important unmet needs. These observations are consistent with previous reports emphasizing the benefits of multidisciplinary and patient-centered care in improving long-term outcomes among individuals with psoriasis [21,22]. By integrating quantitative findings with qualitative narratives, the present study provided a more comprehensive understanding of the psychosocial burden of chronic psoriasis than either method alone. The mixed-methods approach not only quantified the extent of quality-of-life impairment and psychological distress but also explained the personal experiences underlying these outcomes, thereby contributing additional context to the existing literature on psoriasis-related psychosocial health [23].

Strengths and limitations

The principal strength of this study lies in its concurrent mixed-methods design, which enabled integration of quantitative assessment with qualitative exploration of patients' lived experiences, thereby providing a more comprehensive understanding of the psychosocial burden associated with chronic psoriasis. Furthermore, the researcher-developed questionnaire demonstrated satisfactory content validity and internal consistency, supporting its use for assessing the multidimensional outcomes investigated in this study.

Nevertheless, certain limitations should be acknowledged. The study was conducted at a single tertiary care center with a relatively small sample size, which may limit the generalizability of the findings. The cross-sectional design precluded assessment of temporal changes or causal relationships between disease characteristics and psychosocial outcomes. Quantitative findings were based on self-reported responses and may therefore be subject to recall and response bias. Although thematic saturation was achieved during the qualitative component, additional interviews with participants from different healthcare settings and sociocultural backgrounds may have identified additional perspectives on coping strategies and psychosocial adaptation.

## Conclusions

Chronic psoriasis is associated with substantial impairment in quality of life, psychological distress, and adverse effects on social and emotional well-being. By integrating quantitative findings with qualitative patient narratives, the present mixed-methods study provides a more comprehensive understanding of the psychosocial burden experienced by individuals living with chronic psoriasis. The findings highlight the importance of incorporating routine psychosocial assessment, patient education, and appropriate psychological support into comprehensive dermatological care. Future multicenter studies using larger samples and validated patient-reported outcome measures are warranted to further strengthen the evidence and guide patient-centered management strategies.

## Appendices

Section A: demographic information

Age: ______ years

Gender □ Male □ Female

Marital Status □ Married □ Unmarried

Employment Status □ Employed □ Unemployed

Education Level □ Below Secondary □ Secondary or Above

Section B: clinical characteristics

Duration of Psoriasis □ <5 years □ 5-10 years □ >10 years

Clinical Type of Psoriasis □ Chronic Plaque Psoriasis □ Other

Scalp Involvement □ Yes □ No

Nail Involvement □ Yes □ No

Visible Area Involvement (Face, Hands, Neck, or Scalp) □ Yes □ No

Disease Severity (PASI Assessment)

□ Mild (PASI <7)

□ Moderate (PASI 7-12)

□ Severe (PASI >12)

Section C: quality of life

Overall, how much has psoriasis affected your quality of life?

□ Mild impairment □ Moderate impairment □ Severe impairment

Section D: psychological distress

Overall, how much psychological distress has psoriasis caused you?

□ Normal (No distress) □ Mild distress □ Moderate distress □ Severe distress

Section E: impact on daily activities

Has psoriasis affected your social interactions?

□ Yes □ No

Has psoriasis affected your work performance?

□ Yes □ No

Has psoriasis affected your sleep quality?

□ Yes □ No

Has psoriasis affected your physical activity?

□ Yes □ No

Section F: emotional impact

Do you experience embarrassment because of psoriasis?

□ Yes □ No

Do you experience anxiety because of psoriasis?

□ Yes □ No

Do you experience low self-esteem because of psoriasis?

□ Yes □ No

Do you experience depressive feelings because of psoriasis?

□ Yes □ No

Section G: coping strategies

Which coping strategies do you commonly use? (Select all that apply)

□ Social avoidance □ Positive reframing □ Seeking social support □ Acceptance

Section H: patient-reported needs

Which of the following would help you manage your condition better? (Select all that apply)

□ Psychological counseling □ Better disease education □ Support groups □ Holistic care approach

Section I: qualitative interview questions

How has psoriasis affected your emotional well-being?

How has psoriasis affected your social relationships and daily activities?

Have you experienced stigma because of visible skin lesions?

What coping strategies have you used to manage your condition?

What additional support do you believe would improve your quality of life?

## References

[1] Bulat V, Šitum M, Delaš Aždajić M, Lovrić I, Dediol I (2020). Study on the impact of psoriasis on quality of life: psychological, social and financial implications. Psychiatr Danub.

[2] Bhosle MJ, Kulkarni A, Feldman SR, Balkrishnan R (2006). Quality of life in patients with psoriasis. Health Qual Life Outcomes.

[3] Ponikowska M, Vellone E, Czapla M, Uchmanowicz I (2025). Challenges psoriasis and its impact on quality of life: challenges in treatment and management. Psoriasis (Auckl).

[4] Walniczek P, Ponikowska M, Kolarczyk EB, Spaleniak P, Mróz-Kijowska K, Czapla M, Uchmanowicz I (2025). Predictors of quality of life in psoriasis patients: insights from a cross-sectional study. Psoriasis (Auckl).

[5] Agarwal K, Das A, Das S, De A (2022). Impact of psoriasis on quality of life. Indian J Dermatol.

[6] Blackstone B, Patel R, Bewley A (2022). Assessing and improving psychological well-being in psoriasis: considerations for the clinician. Psoriasis (Auckl).

[7] Lee YW, Park EJ, Kwon IH, Kim KH, Kim KJ (2010). Impact of psoriasis on quality of life: relationship between clinical response to therapy and change in health-related quality of life. Ann Dermatol.

[8] Nagarajan P, Thappa DM (2018). Effect of an educational and psychological intervention on knowledge and quality of life among patients with psoriasis. Indian Dermatol Online J.

[9] Meneguin S, de Godoy NA, Pollo CF, Miot HA, de Oliveira C (2020). Quality of life of patients living with psoriasis: a qualitative study. BMC Dermatol.

[10] Luna PC, Chu CY, Fatani M, Borlenghi C, Adora A, Llamado LQ, Wee J (2023). Psychosocial burden of psoriasis: a systematic literature review of depression among patients with psoriasis. Dermatol Ther (Heidelb).

[11] Hewitt RM, Ploszajski M, Purcell C (2022). A mixed methods systematic review of digital interventions to support the psychological health and well-being of people living with dermatological conditions. Front Med (Lausanne).

[12] Armstrong A, Bohannan B, Mburu S (2022). Impact of psoriatic disease on quality of life: interim results of a global survey. Dermatol Ther (Heidelb).

[13] Fredriksson T, Pettersson U (1978). Severe psoriasis - oral therapy with a new retinoid. Dermatologica.

[14] Lynn MR (1986). Determination and quantification of content validity. Nurs Res.

[15] Braun V, Clarke V (2006). Using thematic analysis in psychology. Qual Res Psychol.

[16] Sarkar R, Chugh S, Bansal S (2016). General measures and quality of life issues in psoriasis. Indian Dermatol Online J.

[17] Frede N, Hiestand S, Schauer F (2023). Psoriasis and psoriatic arthritis have a major impact on quality of life and depressive symptoms: a cross-sectional study of 300 patients. Rheumatol Ther.

[18] Jankowiak B, Kowalewska B, Krajewska-Kułak E, Khvorik DF (2020). Stigmatization and quality of life in patients with psoriasis. Dermatol Ther (Heidelb).

[19] Zhong H, Yang H, Mao Z, Chai X, Li S (2021). Impact of moderate-to-severe psoriasis on quality of life in China: a qualitative study. Health Qual Life Outcomes.

[20] Ghezzi G, Costanzo A, Borroni RG (2024). Health-related quality of life in psoriasis: literature review. J Clin Med.

[21] Liu Q, Feng L, Wan C, Tan J, Yu J, Wang L (2022). Development and validation of the psoriasis scale among the system of quality of life instruments for chronic diseases QLICD-PS (V2.0). Health Qual Life Outcomes.

[22] Narayanan S, Guyatt V, Franceschetti A, Hautamaki EL (2015). Disease burden and patient reported outcomes among patients with moderate to severe psoriasis: an ethnography study. Psoriasis (Auckl).

[23] Kouris A, Christodoulou C, Stefanaki C (2015). Quality of life and psychosocial aspects in Greek patients with psoriasis: a cross-sectional study. An Bras Dermatol.

